# Improvement of Tool Steel Powder Cold Sprayability Via Softening and Agglomeration Heat Treatments

**DOI:** 10.1007/s11666-022-01320-4

**Published:** 2022-01-18

**Authors:** D. Poirier, Y. Thomas, B. Guerreiro, M. Martin, M. Aghasibeig, E. Irissou

**Affiliations:** National Research Council of Canada, 75 de Mortagne Blvd., Boucherville, QC J4B 6Y4 Canada

**Keywords:** Cold spray, Tool steel, H13, Powder processing, Heat treatment, Particle velocity

## Abstract

Cold spray can produce deposits from a broad range of materials but reports on cold spray of steels are still limited to the few steel families demonstrating high ductility and medium strength. Softening and agglomeration of steel powders via heat treatment in a rotary tube furnace were investigated as promising ways to improve H13 tool steel powder cold sprayability. By adjusting starting powder size, as well as heat treatment conditions (maximum temperature, cooling rate and heat treatment atmosphere), cold spray of H13 powder improved from virtually no deposition to the production of dense, sound and thick deposits with a powder deposition efficiency of 70%. Powder agglomeration, surface state, microstructure evolution and softening are identified as key factors determining the powder deposition efficiency and resulting deposit microstructure. The developed powder modification method has the potential to facilitate the cold spray of all steels subjected to martensitic transformation.

## Introduction

The cold spray process is a solid-state coating and additive manufacturing technology that uses high-pressure compressed gas to propel micron-sized solid particles onto a substrate under atmospheric conditions at very high deposition rate (Ref 1, 2). Upon impact, the powder particles plastically deform, and, if they reach a critical velocity, adhere to form a continuous deposit allowing producing coatings and near-net shape components without melting the deposited material and with minimal heat input on the substrate. Since the cold spray process has been introduced over 25 years ago (Ref 3), it has been intensively studied for a broad range of materials and the technology available has greatly been improved. However, reports on cold spray of steels, which is the most common engineering material, are still limited to few steel families.

Austenitic stainless steel powders such as 304 and 316 are easily cold sprayed into dense deposits with helium process gas, while deposits with few percent porosity are typically obtained when these powders are sprayed with nitrogen or compressed air (Ref 4-9). These good results are attributed to the high ductility and medium strength of austenitic stainless steels (Ref 10), making them ideal candidates for cold spray. However, very little can be found about cold spray of high strength steels and, in particular, there is no study in the open literature regarding the cold spray of tool steels that are specifically designed to resist deformation. It is well known that cold spraying of hard feedstocks is challenging (Ref 11). With the hardest feedstocks, the maximum achievable coating thickness is limited to a single layer of particles due to the poor deformation of the hard particles impacting the already deposited hard layer (Ref 12, 13). Therefore, development of new strategies that could improve cold sprayability (including deposit quality, deposition efficiency (DE) and deposition rates (DR)) of tool steels and other high strength steels could open the door to new approaches to design changes, repair, and additive manufacturing in the tooling industry, as well as other industries.

One strategy to improve deposit‘s quality is to focus on the cold spray conditions. An increase in particles velocitiy through the use of helium (He) as the propelling gas or through increased propelling gas pressure and/or temperature will increase impact energy, hence particle deformation (Ref 11). An increase in materials temperature (e.g., through powder pre-heating (Ref 14), substrate heating (Ref 15), laser-assisted cold spray (LACS) (Ref 16)) will also facilitate particle deformation. However, there are certain limitations in using these aforementioned approaches. Process costs increase drastically with the use of He. Also, there is ultimately a practical technological limit to the cold spray equipment capabilities in terms of maximum pressure and temperature it can operate. Substrate or powder temperature control, including during LACS, is not straightforward and the heat input may lead to thermal stresses with the possibility of generating cracks and deposit delamination. The increase in temperature can also detrimentally oxidize the substrate surface and/or modify the substrate mechanical properties at the surface due to microstructural changes.

Powder engineering is another approach that presents high potential to enhance cold sprayability of hard materials and improve coating quality. It has been shown that porous WC-based agglomerated powder with relatively low particle strength produces denser and thicker cold spray coatings (Ref 17, 18). The presence of pores reduces the density of the particles that are then easier to accelerate. Also, the loosely agglomerated and porous particles deform more easily upon impact (pseudo-deformation). However, the particle porosity and/or strength levels have to be carefully adjusted to allow deformation at particle impact while preventing particle fragmentation, thus allowing optimized coating deposition. Two other powder engineering approaches, involving careful engineering of reinforcement or matrix phase, have shown interesting results but are limited to composites (Ref 12, 19).

In addition to these methods, hardness of several metallic alloys can be drastically modified through microstructural transformations (Ref 20). However, very few studies have been conducted to adjust alloy powder microstructure to improve the cold sprayability, and these seem to be restricted to Al alloys. In initial attempts, Al alloys powders were homogenized (Ref 21) or homogenized and quenched to obtain a solid solution microstructure (Ref 22-24). Removal of the cast microstructure is shown to improve powder cold sprayability (increase in deposition efficiency and coating quality) and it potentially eliminates the need to solid solutionize the full deposit prior to the aging step of the heat treatment performed to optimize part properties (Ref 22-24). Lately, successfull cold spray of solution heat-treated and aged Al 7075 has been performed. Although this powder was shown to be harder than its quenched counterpart, leading to lower deposition efficiency, resulting coatings were denser and showed indications of improved coating cohesion (Ref 25).

There are thousands of steel grades, each having different compositons and/or heat treatments. Steel powders are sold based on the grade compositions only: Their microstructures and hence their mechanical properties can drastically differ from the bulk grade. The greatest variations in mechanical properties are expected for steel compositions subjected to martensitic transformation, i.e., the transformation of the steel austenite phase to a very hard metastable iron phase supersaturated in carbon when the steel is cooled, or quenched, rapidly enough to prevent carbon diffusion (Ref 20). These steel compositions include carbon steels, tool steels, high strength steels and martensitic stainless steels. Gas or water atomized powders being subjected to very fast cooling rates during their manufacturing (about 10^5^ °C/s for powder sizes of interest for cold spray and produced via gas atomization (Ref 26)), powders of these steel compositions are typically composed of retained austenite and martensite and can be very hard (Ref 25, 27). In theory, these are amenable to significant softening upon phase transformation via annealing heat treatment, potentially making them more deformable during cold spray process. However, little can be found in the literature on the characterisation of steel powder microstructure and hardness, and even less on their modification. One of the reasons for this low interest is that most additive manufacturing technologies involve remelting of the powders, hence the irrelevance of the initial powder state regarding hardness and particle deformability (Ref 28, 29).

Typical annealing temperature for H13 (bulk) is from 845 to 900  °C with a maximum cooling rate of 22 °C/h and similar values are used for other tool steels (Ref 30). At this temperature, which is above the eutectoid temperature, the matrix is transformed to austenite. Since carbides are more soluble in this phase, carbide dissolution occurs. The steel is then cooled down slowly to prevent the formation of hard martensite and to allow the growth of carbides, thus minimizing their strengthening effect. Annealing is already used for H13 powders in conventional powder metallurgy, indicating this is an economically viable method (Ref 31). After shaping, the produced part needs to be heat treated via hardening/tempering stages to raise its hardness to the level required by the application. This development strategy presents the advantage of being simple and requiring readily available equipment.

In this work, we report on a new steel powder modification method to improve H13 powder cold sprayability. Powder annealing with and without agglomeration is investigated using two initial H13 powder size distributions. Heat treatment temperature, cooling rate and atmosphere are varied, and their impact on powder cold sprayability is assessed through microstructure, deposition rate and critical velocity evaluation. The best obtained deposit is heat treated and its hardness after heat treatment is compared with bulk H13.

## Experimental Methodology

### Feedstock Powders

Two commercially available H13 chromium hot-work tool steel powders from Sandvik Osprey Ltd (Neath, Wales, UK) with particle size distributions nominally between 10 and 45 µm and <16 µm were used. Herein, the two powders are named as C-AR and F-AR for the as-received coarse and fine powders, respectively. Given compositions of the two powders are shown in Table 1.

**Table 1:** Chemical compositions of H13 powders (wt.%)

Type	ID	Composition
Coarse +10 -45 µm	C-AR	5.09% Cr, 1.44% Mo, 1.03% V, 0.95% Si, 0.39% Mn, 0.37% C, Fe balance
Fine <16 µm	F-AR	5.20% Cr, 1.70% Mo, 1.07% V, 1.12% Si, 0.33% Mn, 0.47% C*, Fe balance

*Slightly higher carbon content than the maximum allowable for H13 composition (0.47% instead of 0.45%)

### Cold Spray Process

For initial cold spray trials, rectangular low carbon steel 1020 substrates with dimensions of 76.2 × 76.2 mm and thickness of 3.2 mm were used, while for deposition of the coatings intended for post-cold spray process heat treatment (section 4 of the results), N type Almen strips of SAE 1070 steel were used as the substrates. Prior to the coating depositions, the substrates were degreased using ethanol and then manually grit-blasted with an air pressure of 0.276 MPa (40 psi) at an approximate blasting distance of 150 mm at 30°, using grit 24 (~975 µm) alumina (Al_2_O_3_). In addition, an air gun was used to remove extraneous alumina particles from the grit-blasted surface.

Deposition of coatings was performed using a PCS-1000 cold spray system (Plasma Giken, Tokyo, Japan) equipped with a commercial tungsten carbide converging-diverging nozzle PNFC2-010-30S. Nitrogen was used as the propellant gas and the process parameters are specified in Table 2.

**Table 2:** Cold spray process parameters

Gas T (°C)	Gas P (MPa)	SOD (mm)	Robot traverse speed (mm/s)	Powder feed rate (g/min)	# passes
950	4.9	45	100 or 300	15-32	5 or 10

SOD: Standoff distance

Deposition rates were obtained from the measured coating thickness divided by the number of passes and were normalized for a powder feedrate of 20 g/min and a traverse speed of 300 mm/s. The deposition efficiency of one powder (F-HT 875 °C-4) has been obtained from the ratio of the weight of the coating, measured by weighing the samples before and after spraying, to the weight of the powder that is sprayed toward the substrate obtained from the measured powder feed rate and the calculated spraying time. DEs of the other powders were calculated from a proportionality rule using their normalized deposition rates and DE of F-HT 875 °C-4 powder as a reference.

### Heat Treatment

Two different powder heat treatment setups were studied, namely powder tempering and powder annealing. Powder tempering was performed in a 100 mm diameter standard (i.e., static) quartz tubular furnace (Lindberg, Riverside, MI, USA) under nitrogen. Powder was held at 600  °C for 4 h under nitrogen and furnace cooled. The typical annealing temperature for H13 (bulk) being at 845-900  °C, i.e., above the temperature at which significant sintering can occur, annealing was performed in a 100 mm rotary quartz tube furnace (model OFT1200X-4-R-IL-UL, MTI Corporation, Richmond, CA, USA) to limit powder agglomeration. The annealing maximum temperature, held for 2 hours, was fixed at 875 °C, while the heat treatment cooling rate and heating atmosphere were varied (22 °C/h, 70 °C/hr or 350 °C/hr and nitrogen or high-purity (HP) argon, respectively).

Selected cold spray coatings were heat treated in the standard quartz tubular furnace under nitrogen atmosphere at 1200 °C for 3 h (austenitizing and sintering) followed by water quenching and two steps of martensite tempering at 570 °C for 2 h for improved ductility.

### Materials Characterization

The average particle size and the volume-weighted particle size distribution were measured via a laser diffraction particle size analyzer (LS320, Beckman Coulter, Miami, FL, USA).

Deposited coatings were cut perpendicular to the spraying direction. The sectioned samples and the powders were cold vacuum mounted in an epoxy resin and polished according to the standard metallographic preparation procedures using a 0.25 µm colloidal silica suspension for the final polishing step. To reveal the microstructure of the polished samples, Vilella’s reagent (100 ml ethanol, 5 ml hydrochloric acid and 1 g picric acid) and Nital (2 ml HNO_3_ and 98 ml ethanol) were used as etchants for powder and cold spray samples, respectively. A field emission gun scanning electron microscope (FEG-SEM S-4700, Hitachi, Tokyo, Japan) was used for characterization of the feedstock powder morphologies in secondary electron imaging mode (SE) and for cross-sectional microstructural analysis of the powders and cold spray coatings before and after heat-treatments in SE or backscattered electron imaging (BSE) modes.

Nanohardness of the powders was evaluated by a MTS Nano Indenter-G200 (Oak Ridge, TN, USA) with a Berkovitch tip under a load of 3 gf. The average nanohardness values were obtained from at least 15 indentations performed on the polished cross sections of the powders. Hardness of a selected heat-treated coating was measured using a Rockwell C hardness tester (model B2000, Wilson Instruments, now Buehler, Lake Bluff, IL, USA) by averaging 5 indentations taken on the ground surface of the >2 mm thick coating. Powder phases were identified using XRD (Bruker D8 Discover x-ray diffractometer, Madison, WI, USA) with Cu-Ka1 radiation at 1.6 kW. A step size of 0.02° was selected for a range of 10-90°.

### Simulations of Particle Impact Velocities

Particle velocity at impact was simulated using the 3D computational fluid dynamics (CFD) module of CSAM Digital Solutions Software (NRC, Canada). Details regarding the governing equations, their numerical discretizations, as well as particle tracking, used in this software can be found in a previous publication (Ref 32). The simulations were performed for a gas temperature of 950 °C, gas pressure of 4.9 MPa, standoff distance of 40 mm, and assuming dense spherical particles.

## Results and Discussion

### Coarse H13 Powder Heat Treatment and Coating Properties

The effect of tempering and annealing coarse H13 powder on the agglomeration and softening was investigated. The tempering (at 600 °C) and annealing (at 875 °C) heat treatments were performed under nitrogen. In the case of annealing, slow cooling rate of 22 °C/h was maintained until 500 °C, and then, the powder was furnace cooled; for the tempering, only furnace cooling was used.

**Fig. 1:**
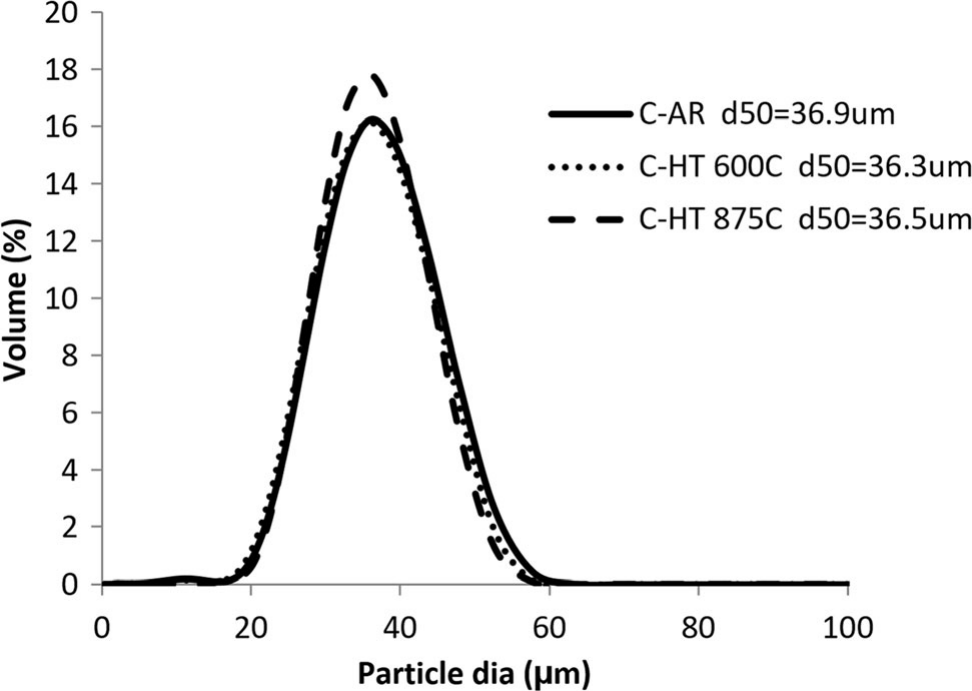
Particle size distribution of coarse powder (10 to 45 µm), As-Received, and heat-treated (C-AR: As-Received, C-HT 600 °C: heat-treated at 600 °C, C-HT 875 °C: heat-treated in a rotary tube furnace at 875 °C)

Figure 1 displays the particle size distributions in as-received condition (C-AR) and after the two different heat treatments (C-HT 600 °C and C-HT 875 °C) for the coarse powder. Powder caking occurred after HT 600 °C even at the selected relatively low temperature and crushing was required to recover the powder. Agglomeration was however less pronounced after C-HT 875 °C performed in the rotary tube furnace and only a simple mixing in a V-blender was sufficient to break the soft agglomerates and recover most of the powder. Sieving to <45 µm still had to be performed to remove the coarse tail which is not seen on Fig. 1.

**Fig. 2:**
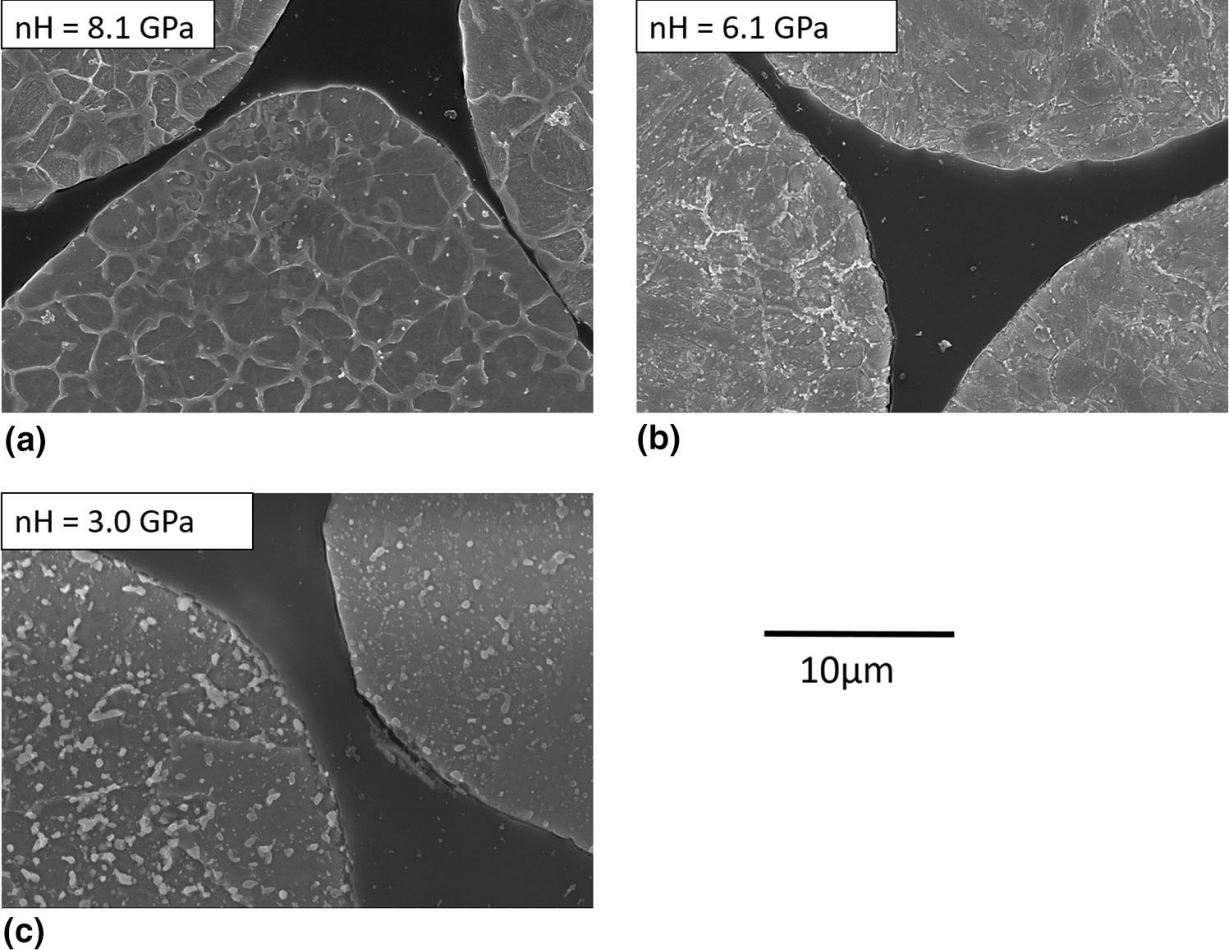
Etched microstructure (SE, 15kV, 5000X) and nanohardness of the coarse powder (a) C-AR, (b) C-HT 600 °C and (c) C-HT 875 °C

Figure 2 shows the microstructures and nanohardness of the heat treated coarse powder at the two conditions compared to the as-received powder. A network surrounding darker grains is present for the as-received powder microstructure. The observed grains are mainly martensite with some retained austenite due to the fast cooling rate during manufacturing (Ref 31) and as confirmed via XRD (results not shown here). The network is composed of carbides, as typically observed for tool steel as-cast microstructures obtained under high cooling rates; it is the result of microsegregation during the solidification process (Ref 33, 34). The high powder hardness of 8.1 GPa is therefore attributed to the combined effect of the hard martensite, the soft retained austenite and the hard carbide skeleton. When converted to HRC (about 56 HRC (Ref 35)), this hardness value is very close to those obtained for quenched bulk H13 (51-54 HRC, (Ref 36)).

Powder microstructure after C-HT 875 °C is composed of spheroidized carbides, the desired morphology to lower hardness and maximize formability. The carbides have precipitated and grown during the slow cooling step of the heat treatment. Owing to the slow cooling rate, martensite formation has also been prevented—grains are most probably composed of ferrite (Ref 7), hence the low hardness value of 3.0 GPa. Powder microstructure after C-HT 600 °C is at an intermediate stage between the as-received and the fully annealed powder microstructures. The temperature of this heat treatment is not high enough for austenitisation but martensite tempering can occur and carbides can slightly evolve. The associated hardness is 6.1 GPa.

**Fig. 3:**
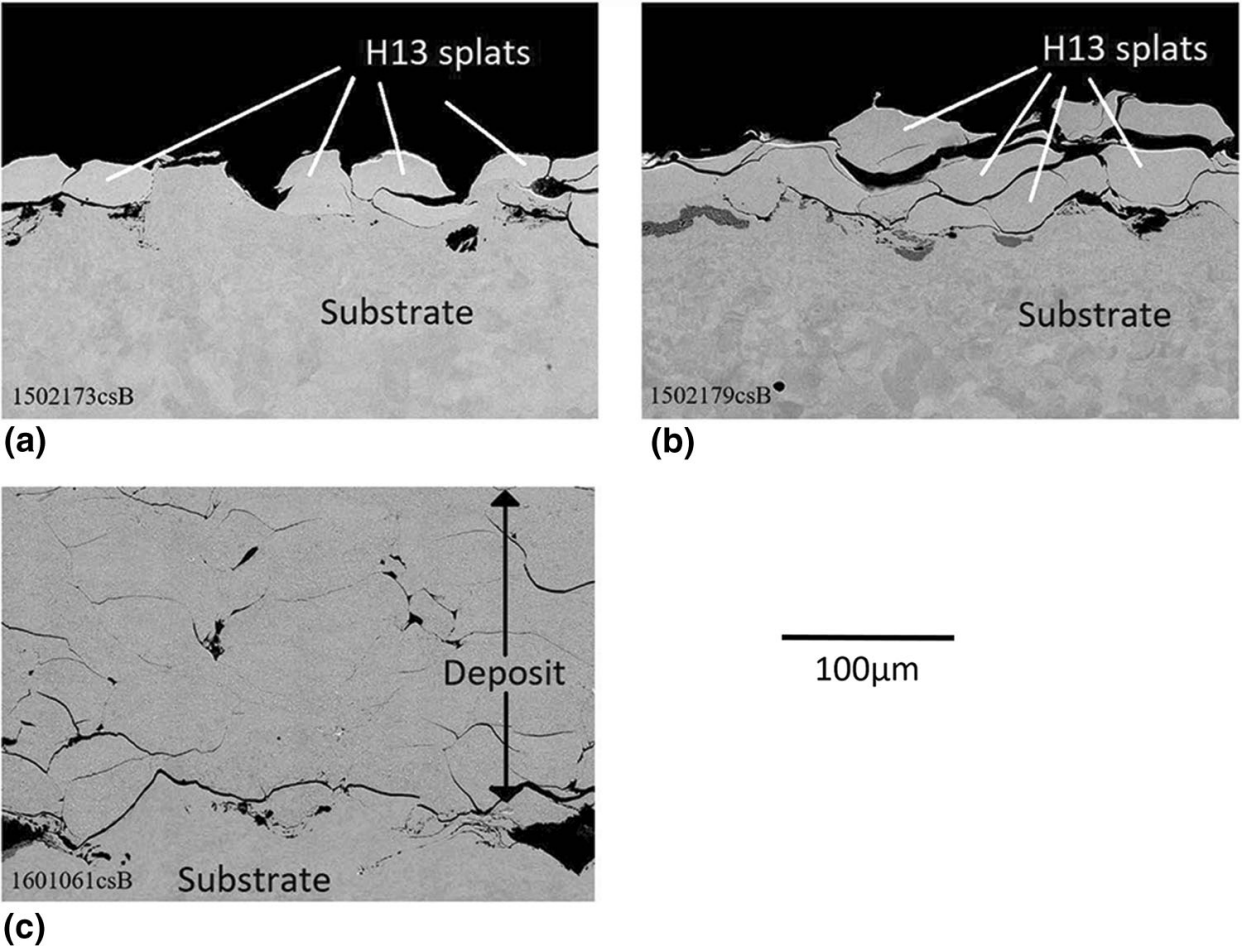
Microstructures (BSE, 15kV, 500X) of deposits produced with H13 coarse powder (a) C-AR (b) C-HT 600 °C (c) C-HT 875 °C

Coarse powder lots were cold sprayed in as-received condition and after heat treatments. The resulting coating microstructures can be seen on Fig. 3. As expected, only a single layer of particles could be deposited when using the hard, as-received H13 powder, i.e., it was not possible to deposit C-AR on the deposited C-AR first layer. The heat treatment at 600 °C slightly improved powder cold sprayability but the resulting coating is still limited to a thin layer and displays poorly bonded particles on a non-uniformed second layer. The coatings prepared with powder HT at 875 °C and sieved at <45 µm, however, were thick but contained cracks as shown in Fig. 3(c).

These tests demonstrated that H13 powder heat treatment to soften the powder is possible without too much sintering if powders are agitated at high temperature—for instance in a rotary tube furnace. Annealing heat treatments provided a powder with better cold sprayability than the tempering heat treatment due to a powder hardness reduction of more than 60%. However, the resulting coating still presents poor interparticle bonding. The annealing heat treatment was selected for further testing with the fine powder.

### H13 Fine Powder Modification (Heat Treatment and Agglomeration) and Coating Properties

#### Initial Trials

Effect of powder agglomeration and softening on cold sprayability of H13 is investigated. In order to obtain an agglomerated powder of an appropriate size for cold spray, powder heat treatment was performed using the fine powder (F-AR) with particle size of <16 µm. Considering the powder caking obtained out of the standard quartz tubular furnace with the coarse powder, only the rotary tube furnace was used to perform the annealing treatment HT 875 °C. The powder was then sieved at <45 µm to remove the tail of coarser agglomerates. This powder is identified as F-HT 875 °C-1a throughout this manuscript.

**Fig. 4:**
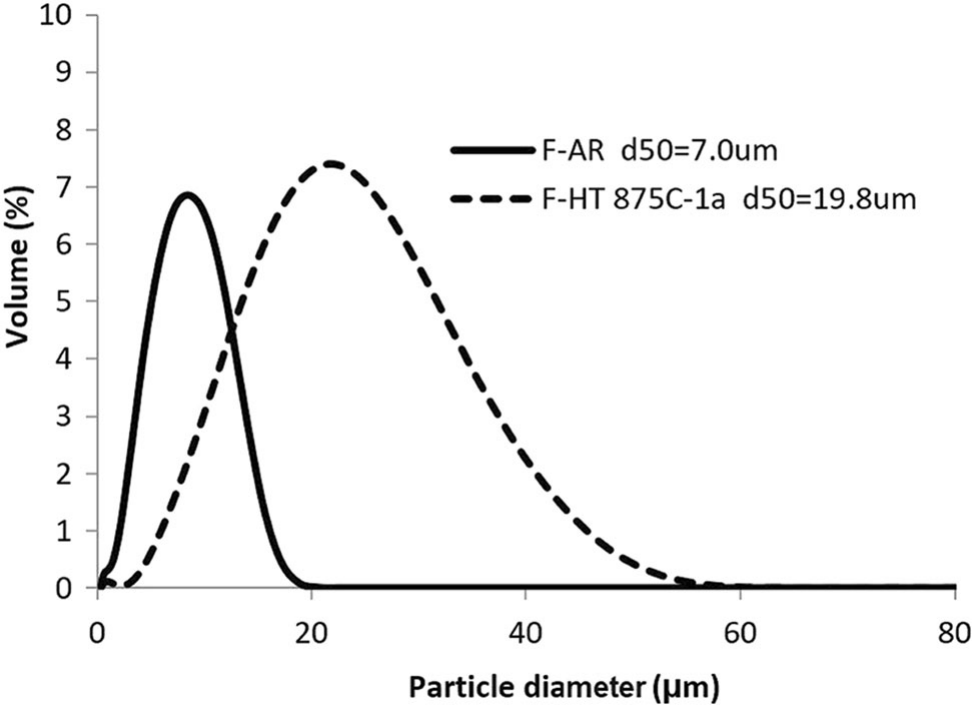
Particle size distribution of the fine H13 powder, As-Received (F-AR) and HT 875 °C under nitrogen atmosphere (F-HT 875 °C-1a)

**Fig. 5:**
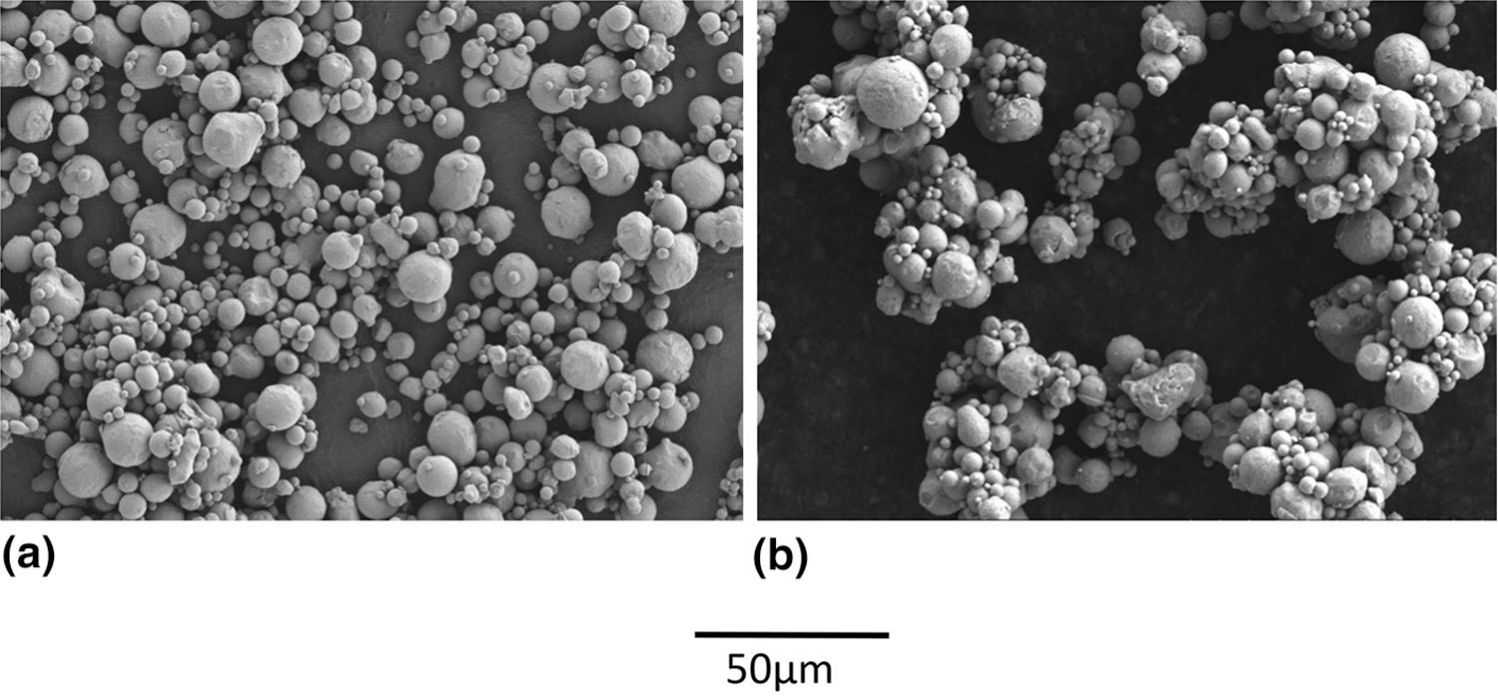
Fine H13 powder (SE, 15kV, 1000X) (a) before (F-AR) and (b) after agglomeration by HT 875 °C (F-HT 875 °C-1a)

As shown in the particle size distributions of Fig. 4, agglomeration was readily obtained with the HT and d_50_ was increased from 7 to 20 µm, which is an appropriate size for cold spray. This agglomeration is also visible when observing the powder using SEM (Fig. 5b). The d_50_ of the agglomerates is still smaller (near half) than the size of the coarse powder.

**Fig. 6:**
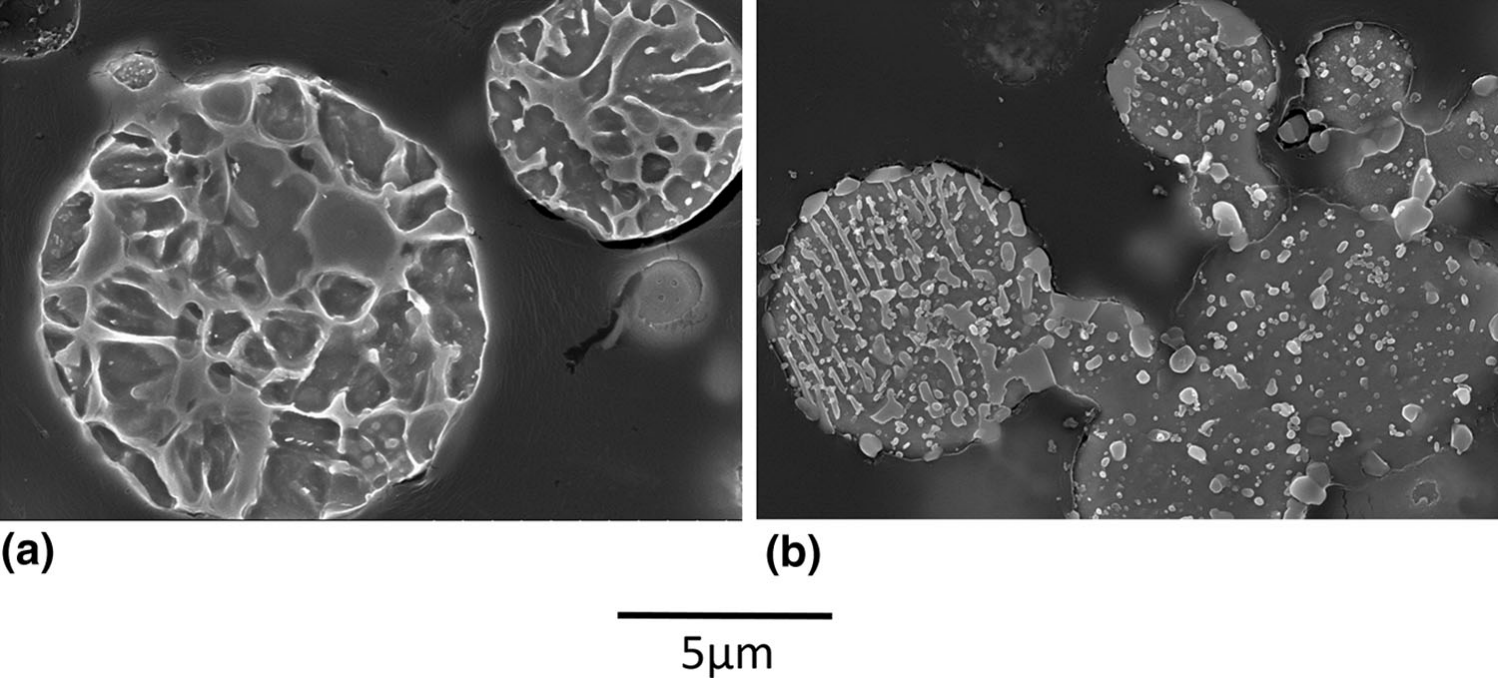
Etched microstructures (SE, 15kV, 10,000X) of fine H13 powder (a) F-AR (b) F-HT 875 °C-1a

The powder microstructures obtained before and after HT are similar to those obtained for the coarse powder (Fig. 6 versus Fig. 2). Similar to the coarse powder, heat treatment at 875 °C has also spheroidised the carbides of the fine lot, without any visible decarburization at the powder surface. However, unlike the coarse powder, interparticle bonding was clearly observed with F-HT 875 °C-1a, as shown in Fig. 6(b). It is believed that the fine lot is more prone to diffusion and sintering than the coarse one due to its higher surface area (Ref 37). Due to the small powder size, hardness of the particles could not be reliably measured, although it is expected that a significant hardness reduction is obtained after the heat treatment similar to what was observed for the coarse powder.

**Fig. 7:**
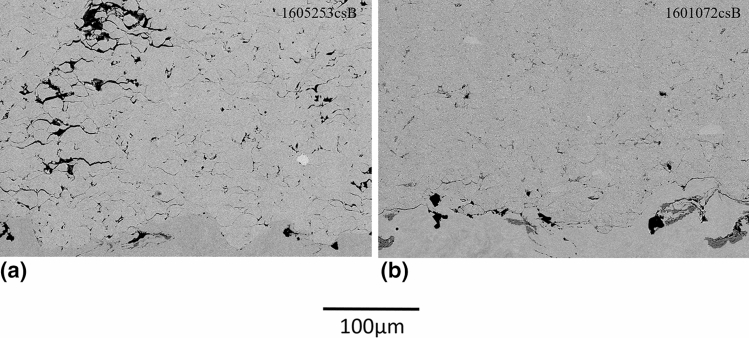
Microstructure (BSE, 15kV, 500X) of deposits produced with H13 fine powder (a) as-received (F-AR) and (b) F-HT 875 °C-1a

This fine powder was cold sprayed in as-received condition and after heat treatment. The corresponding coating microstructures can be seen in Fig. 7. In comparison with the coating deposited using the as-received coarse powder and limited to a monolayer, a thicker coating with a normalized deposition rate of 33 µm/pass was achieved when spraying the fine powder. The coating demonstrates several cracks within the microstructure exhibiting poor adhesion in between the deposited particles (Fig. 7a). The coating quality was improved for the heat treated powder (F-HT 875 °C-1a) as shown in Fig. 7(b). The normalized deposition rate of this powder was 78 µm/pass compared to 42 µm/pass for the C-HT 875 °C powder; almost twice higher deposition rate is obtained with the fine powder heat treated under the same conditions.

#### Optimization of Powder Modification Process (Powder Treatment Cooling Rate and Atmosphere)

Two parameters were specially studied for the optimization of the powder modification process with the fine powder: increase in cooling rate to shorten heat treatment duration, and heat treatment atmosphere. For these trials and the following, the robot traverse speed during spraying was also decreased to 100 mm/s. Powder F-HT 875 °C-1b was heat treated at the same condition as for F-HT 875 °C-1a. However, a smaller particle size distribution was obtained for this powder (d_50_: 17.2 µm versus 19.8 µm). A similar deposition rate (normalized for powder feedrate and traverse speed) was obtained for these two powders that were sprayed at different traverse speeds (78 and 74 µm/pass).

**Fig. 8:**
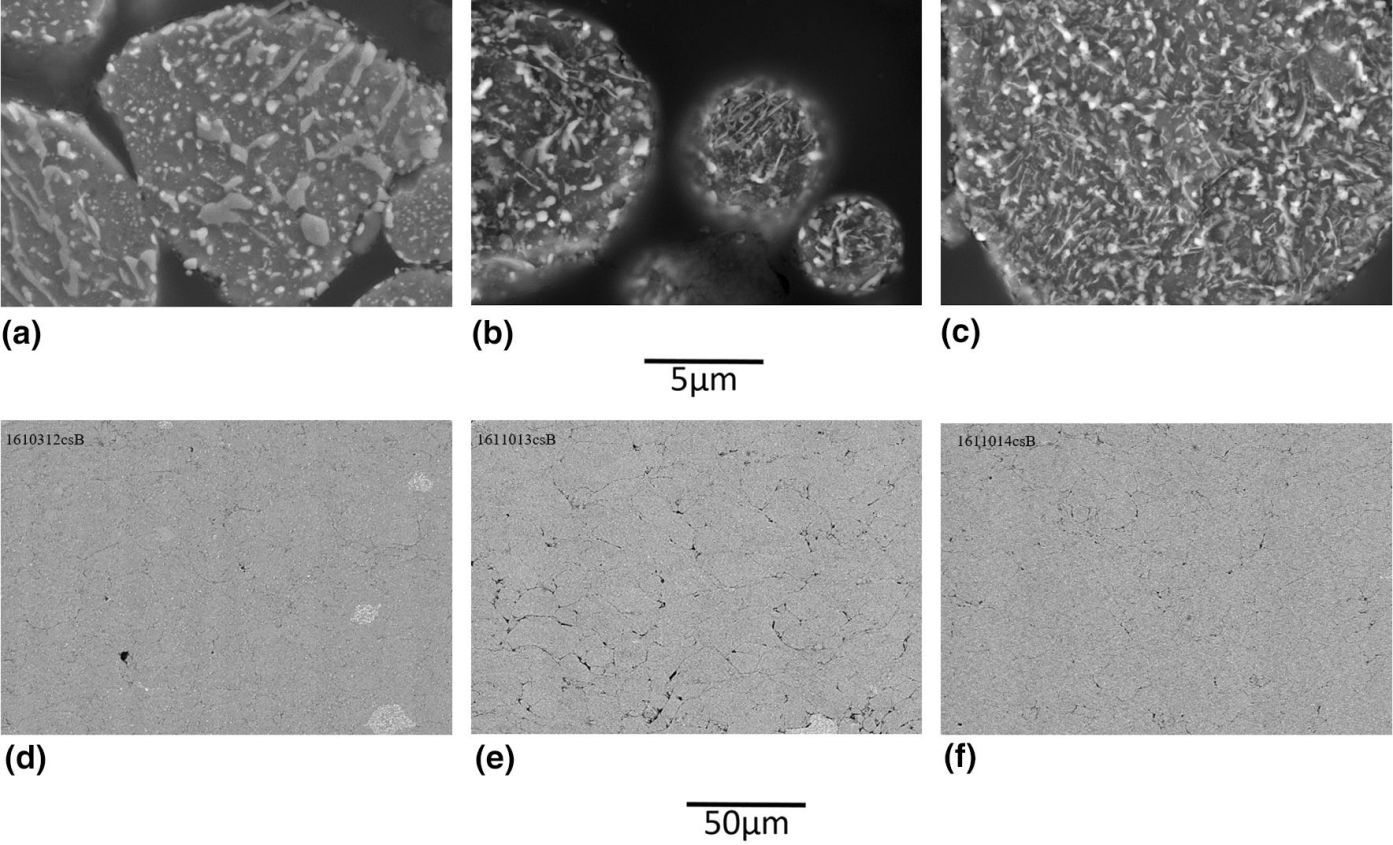
Microstructures (SE, 15kV, 10,000X) of fine H13 powders after HT 875 °C under various cooling rates and the resulting coating microstructure (BSE, 15kV, 1000X) (a-d) 22 °C/hr (F-HT 875 °C-1b) (b-e) 70 °C/hr (F-HT 875 °C-2) (c-f) 350 °C/hr (F-HT 875 °C-3)

In regards to the cooling rate, it is to be noted that the value of 22 °C/h for the cooling rate of bulk H13 is fixed not only to avoid martensite formation but also to control other material aspects which are irrelevant for powder heat treatment, such as heat transfer through thick parts or part cracking/distortion. The continuous cooling transformation (CCT) phase diagram of H13 can help to predict maximum cooling rate allowable prior to significantly increasing powder hardness. According to Dobrzański et al. (Ref 38), it appears that the cooling rate can be increased up to about 70 °C/hr without any significant change in hardness/microstructure, whereas cooling rates above are predicted to increase powder hardness drastically (about 3 times) due to the formation of bainite and/or martensite. Fig. 8 compares the powder and coating microstructures for three cooling rates: the initial 22 °C/hr (F-HT 875 °C-1b), 70 °C/hr (F-HT 875 °C-2) and 350 °C/hr (F-HT 875 °C-3). Surprisingly, the coating quality achieved with a powder cooled down at 350 °C/hr is nearly equivalent to the ones obtained with a powder cooled down at 22 or 70 °C/hr. However, the deposition rates were decreased at increased cooling rates (49 µm/pass for 70 °C/hr and 54 µm/pass for 350 °C/hr versus 74 µm/pass for 22 °C/hr). This could indicate a less efficient powder softening, but not to the extent expected by a ferrite-to-martensite transition. According to Fig. 8(e), powder cooled down at 350 °C/hr (F-HT 875 °C-3) possibly presents more lenticular features than slower cooling rates but the complex microstructures do not allow to clearly ascertain differences between the different lots. Preliminary nanoindentation trials on the powders were not successful in measuring powder hardness: The small powder size led to a bias in the measurements in the epoxy. However, the semi-quantitative values were found to be very similar among the three cooling rates. It is hypothesised that the lower austenitisation temperature used here compared to Dobrzański leads to lower carbide dissolution. A lower carbon content in the austenite matrix upon quenching is known to decrease steel hardenability, hence allowing to increase the cooling rate beyond 70  °C/hr without too much hardening of the powder. The heat treatment cooling rate was increased to 350 °C/h for subsequent trials. By doing so, the heat treatment duration was decreased by 3-4 times, which is an important gain in term of productivity. In future work, further cost reduction could be envisioned via the replacement of the expensive gas atomised powders with irregular (cheaper) powders, obtained for example with water atomization. Moreover, powder manufacturing costs could be lowered by using a bigger rotary furnace, hence increasing the powder batch size, or by converting the process to continuous operation. Generally speaking, the increase in powder manufacturing costs associated with the powder modification method can be easily justified in the cases where it leads to a substantial increase in powder deposition efficiency and coating quality, with reduced powder losses, as well as cold spray process times.

**Fig. 9:**
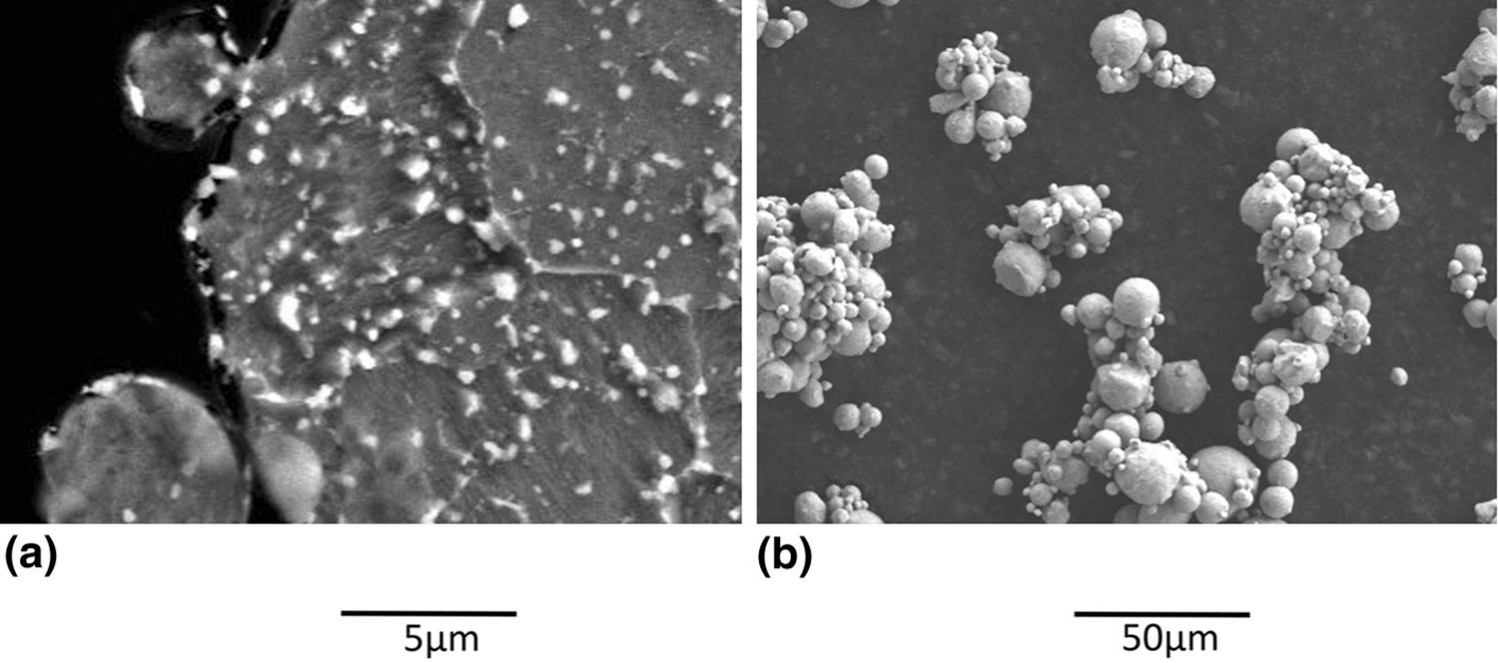
Fine H13 powder after agglomeration by HT 875 °C in Ar (F-HT 875 °C-4) (SE, 15kV) (a) Etched microstructure (10,000X) (b) Morphology (1000X)

**Fig. 10:**
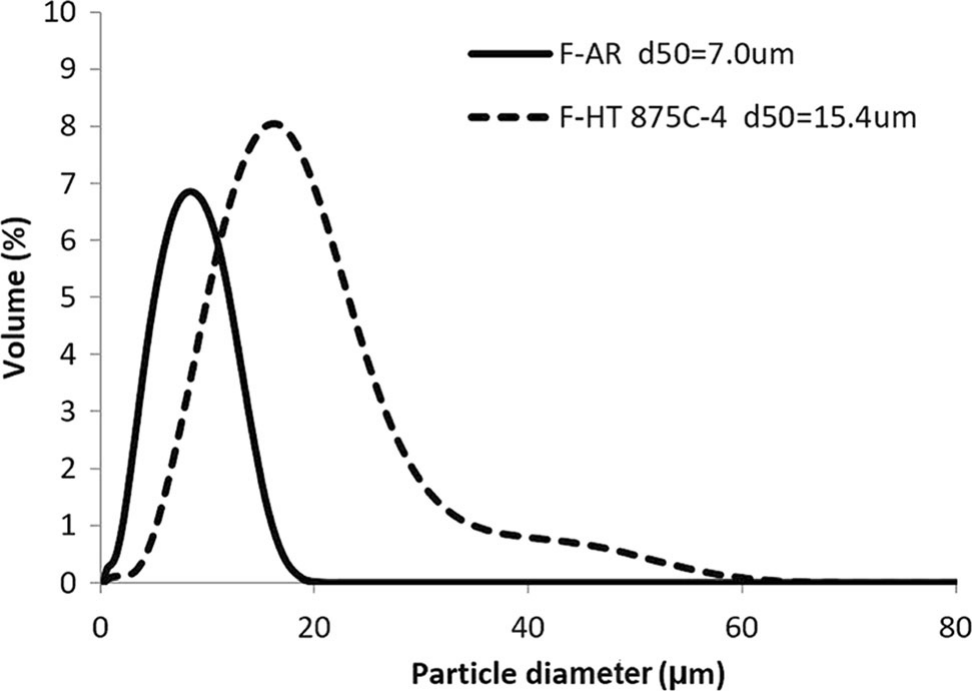
PSD of fine H13 powder before (F-AR) and after HT 875 °C under argon atmosphere and cooled at 350 °C/h in pure argon (F-HT 875 °C-4)

**Fig. 11:**
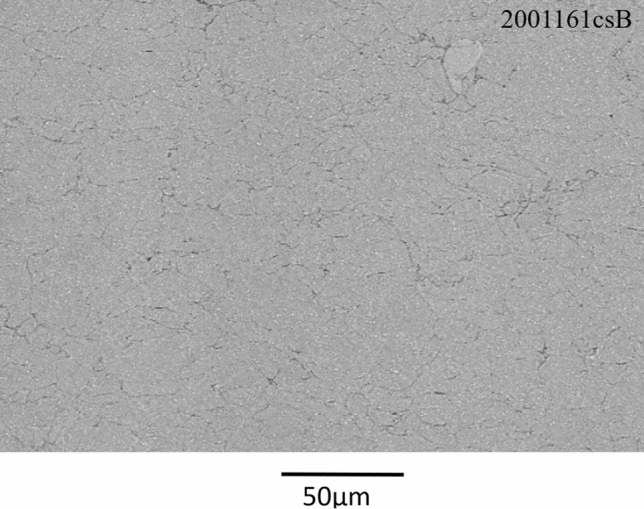
Microstructures (BSE, 15kV, 1000X ) of a H13 deposit sprayed using F-HT 875 °C-4 powder (HT under argon atmosphere and cooled at the rate of 350 °C/h)

A better control of heat treatment atmosphere oxygen level was provided by the use of HP argon (5 ppm O_2_ max) atmosphere instead of nitrogen and an improved tracking and suppression of rotary furnace leaks. These changes did not result in a significant difference in powder microstructure (Fig. 9a) but semi-quantitative EDX analysis at the surface of the particles has shown lower particle surface oxygen content. Extensive powder agglomeration was obtained due to lower powder oxidation favoring sintering. In order to remove the powder from the tube and break the largest agglomerates, a pestle and mortar had to be used, resulting in a decrease in the final agglomerate size to d_50_ = 15.4 µm (Fig. 9b and 10). Herein, this powder is identified as F-HT 875 °C-4. A drastic improvement in coating uniformity as shown in Fig. 11 was obtained with the use of HP Argon atmosphere. The deposition rate was also increased with normalized value of 127 µm/pass yielding to a measured deposition efficiency (DE) of 70%. This is attributed to the minimization of powder oxidation during the heat treatment. A thinner surface oxide layer is known to facilitate the powder cold spray deposition and particle bonding (Ref 39, 40).

**Fig. 12:**
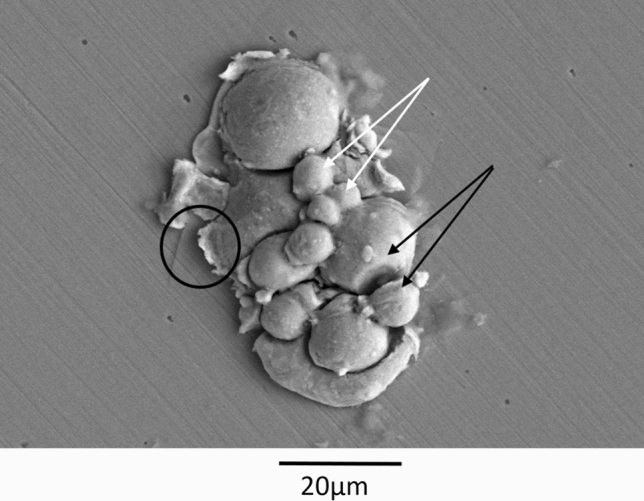
Splat observation of F-HT 875 °C-4 particles on polished mild steel (SE, 15kV, 2500X). Black circle: particle deformation upon impact. White arrows: strongly bonded particles. Black arrows: Particles that have moved with respect to each other upon impact

Splat tests performed with the F-HT 875 °C-4 powder on polished mild steel (Fig. 12) show that agglomerate bonding is sufficiently good to hold particles together in the gas jet up to the impact. Upon impact, indications of individual particle deformation for the largest particles can be observed. While some particles are still strongly bonded together, other particles have moved with respect to each other upon impact. This is indicative of “pseudodeformation” that could favor powder cold sprayability (Ref 17, 18).

### Assessment of Critical Velocity Variation as a Function of Powder Size and with Powder Heat Treatment

Critical velocity is the minimum velocity required to attain particle deposition on the surface, and it is a function of material properties, particle size and process parameters (Ref 41). It is a key parameter widely used in the field of cold spray for understanding coating deposition and optimization. To better understand the effect of powder heat treatment on powder deposition and coating quality, impact velocity of each powder was compared with the critical velocity. In the absence of reliable input powder mechanical property values such as tensile strength, critical velocity calculation using theoretical equations such as in reference (Ref 42) would yield uncertain results. In this study, method introduced by Irissou et al. (Ref 43) was selected as a reliable technique to estimate the critical velocity of each powder. Particle impact velocities were simulated using CSAM Digitals Solutions software and are presented in the next section. Details regarding the calculation of the critical velocity and discussion on the effect of the employed heat treatments on the critical velocity, and thus particle deposition, is then provided in the subsequent section.

#### Simulations of Particle Impact Velocity

**Fig. 13:**
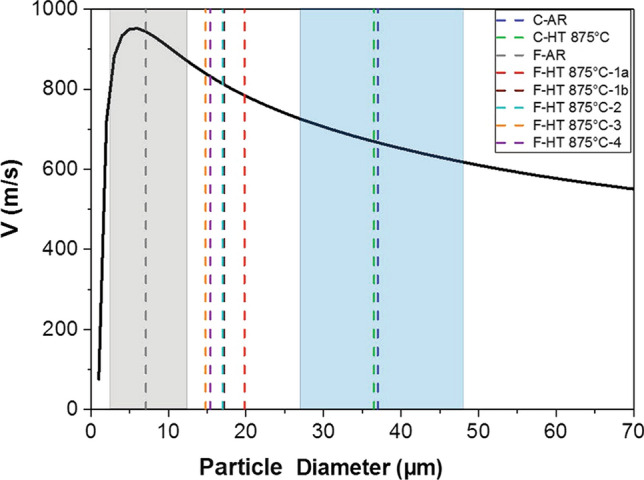
Simulated impact velocities as a function of H13 particle diameter. The gray and blue regions show the particle size range (d_10_ to d_90_) for the as-received fine and coarse powders, respectively. The dashed lines show the d_50_ of different as-received and heat-treated powders

Particle velocity at impact for each particle diameter was simulated using the CSAM Digital Solutions software and the volume weighted average values for each powder batch were computed from the simulation results. Fig. 13 shows impact particle velocity as a function of diameter. The size ranges of the as-received fine and coarse powders are illustrated by the gray and blue regions, respectively, and d_50_ of the different powders is indicated by dashed lines. For particles with diameter >5 µm, the impact velocity decreases with increase in the particle size. The fine tail of the graph with particle size <5 µm corresponds to the particles that are slowed down and deflected by the bow shock formed at the impingement zone. One of the key benefits of agglomeration is to bond these finer particles that do not deposit into larger agglomerates, so they can reach the sprayed surfaces and deposit as demonstrated in Fig. 12. It is worth mentioning that for the agglomorated fine powders exhibiting irregular morphologies, the impact particle velocity is expected to be higher than the simulated value of Fig. 13 due to higher drag coefficients (Ref 44). However, the results provide a valid semi-quantitative approach that allows drawing the conclusions further presented although absolute values should be considered understimated.

#### Calculation of the Critical Velocity

**Fig. 14:**
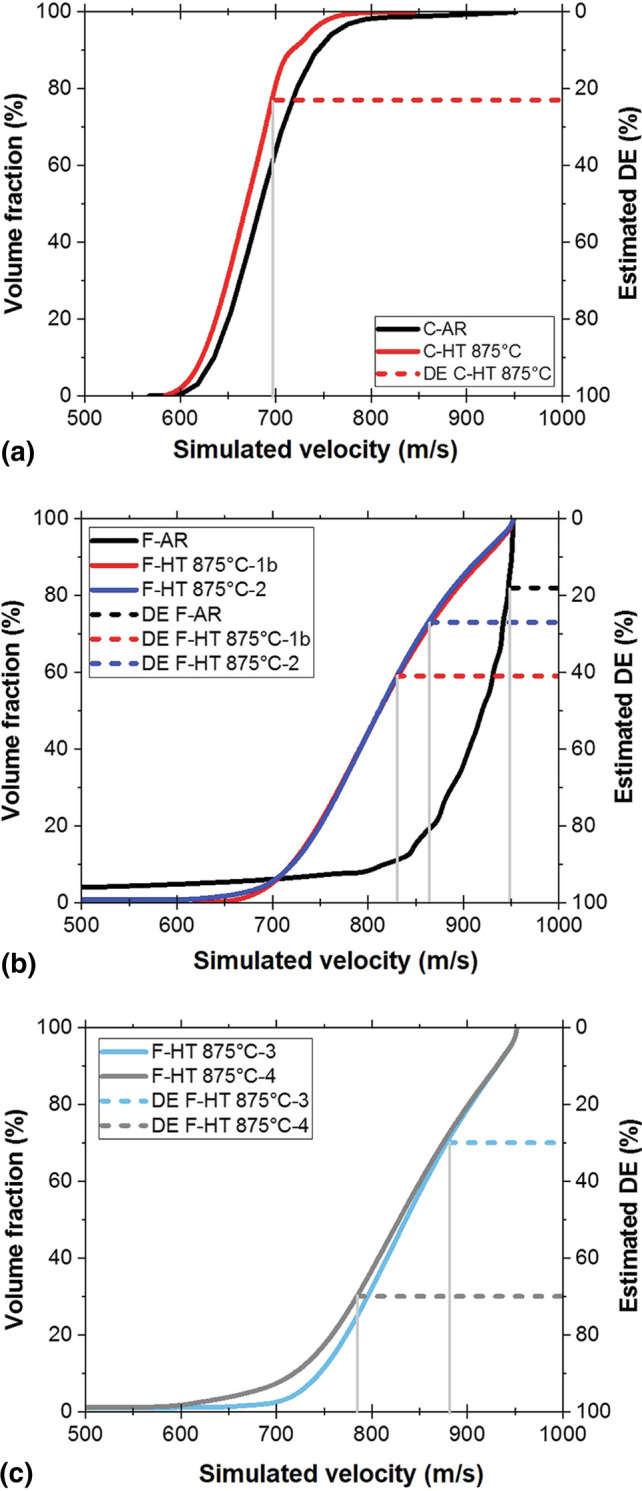
Cumulative volume fraction and calculated DE against simulated velocity for as-received and heat-treated powders (a) C-AR and C-HT 875 °C, (b) F-AR, F- HT 875 °C-1b and F-HT 875 °C-2, and (c) F- HT 875 °C-3 and F- HT 875 °C-4

The critical velocity of each powder was determined from the cumulative volume fraction and deposition efficiency against simulated velocity curves. The impact velocity was simulated for each particle diameter that was obtained from the particle size analysis. The cumulative volume fraction of the particles with velocities lower than each of the simulated velocities was then calculated having the volume fraction of each particle diameter from the particle size analysis, and plotted as shown in Fig. 14. Given the deposition efficiency of each powder (Table 3), the critical velocity was therefore graphically estimated as shown in Fig. 14. For example, in Fig. 14(a), given that powder C-HT 875 °C deposition efficiency is 23%, 77 vol.% of the particles have a velocity below the critical velocity and do not deposit. Therefore, from the intercept point of the dotted red-line (23% DE or 77% cumulative volume fraction) with the velocity curve, one can deduce that the estimated critical velocity is at 698 m/s. The obtained estimated critical velocity values for all the powders are calculated using the same methodology (Fig. 14a to c). Since the simulated velocities of agglomerated powders are underestimated, the estimated critical velocities calculated from these simulated velocities are also underestimated. Only powders with similar size distribution and shape (spherical or agglomerated) present similar particle velocities and can be directly compared; a lower estimated critical velocity would indicate a higher deposition efficiency due to powder softening.

**Table 3:** Normalized deposition rate (DR), estimated DE, eimulated velocity associated to d_50_ (*V*_d50_), simulated average velocity (*V*_avg_), calculated critical velocity (*V*_cr_) and coating quality of as-received and heat-treated powders

ID	HT atm.	HT CR (°C/h)	d_50_ (µm)	Normalized DR (µm/pass)^1^	Estimated DE (%)	V_d50_ (m/s)	V_avg_ (m/s)	V_cr_ (m/s)	Coating quality
C-AR	NA	NA	36.9	0^2^	0	666	680	>850	Almost no deposition
C-HT 875 °C	N_2_	22	36.5	42	23	669	680	698	Cracked coating
F-AR	NA	NA	7	33	18	944	881	950	Cracked coating
F-HT 875 °C-1a	N_2_	22	19.8	78	43	783	790	800	Sound coating
F-HT 875 °C-1b	N_2_	22	17.2	74	41	811	823	830	Sound coating
F-HT 875 °C-2	N_2_	70	17	49	27	813	819	865	Sound coating
F-HT 875 °C-3	N_2_	350	14.8	54	30	836	839	880	Sound coating
F-HT 875 °C-4	HP Ar	350	15.4	127	70^3^	830	828	785	Sound coating

Velocities of the agglomerated powders are underestimated and can not be compared with the values for non-agglomerated powders.

^1^normalized for a feed rate of 20 g/min and traverse speed of 300 mm/s.

^2^particles could not be deposited beyond the first monolayer after multiple passes.

^3^calculated from the ratio of the weight of the coating to the weight of the powder sprayed on the substrate as described in section Cold Spray Process.

Table 3 summarizes the normalized deposition rate (DR), the estimated deposition efficiency (DE), the simulated impact velocity associated to *d*_50_ (*V*_d50_), the simulated average impact velocity (*V*_avg_), the critical velocity (*V*_cr_) and coating quality of all as-received and heat-treated powders investigated in this study. The coarse powders in the as received condition and after heat-treatment have similar particle size (*d*_50_~37 µm) and therefore the same average particle impact velocity (*V*_avg_) of 680 m/s. As C-AR could not be deposited (DE~ 0%), the critical velocity is expected to be higher than the highest particle velocities of this powder, and it conservatively considered as >850 m/s (Fig. 14a). The higher DE of C-HT 875  °C (23%), which is indicative of easier powder deposition, corresponds to a much lower critical velocity (about 700m/s) when input in Fig. 14(a). This is similar to commonly sprayed alloys such as titanium and SS316, (Ref 42) and well within the range of cold spray equipment capability.

For the fine agglomerated powders F-HT 875 °C-1b and F- HT 875 °C-2, where the particle size distributions and consequently the average particle velocities are similar (~820 m/s), the critical velocity increased from 830 m/s to 865 m/s when the cooling rate was increased from 22 to 70 °C/h (F-HT 875 °C-1b versus F- HT 875 °C-, Fig. 14b). This decrease in critical velocity is indicative of a less efficient powder softening at higher cooling rates, which was also illustrated by decrease in the deposition rate in 3.2.2. Since F-HT 875°-C3 has a finer particle size (d_50_: 14.8 µm) than F- HT 875 °C-1b and 2, its average particle velocity is higher at 840 m/s. The higher estimated critical velocity of this powder (880 m/s) is due to the finer particle size and possibly less efficient powder softening. F-HT 875 °C-3 and F-HT 875 °C-4 powders heat-treated at the same condition but in different atmosphere have similar d_50_ values and average velocities (~830-840 m/s). However, the estimated critical velocity of F-HT 875 °C-4 is about 100 m/s lower (Fig 14c). This highlights the positive effect of powder heat-treatment under HP argon atmosphere and thus minimized powder oxidation. Although the absolute value of velocities and therefore estimated critical velocities are likely underestimated as explained before, this conclusion on the effect of the heat treatment conditions based on the analysis of particle dynamics is still valid. These results suggest that further improvement in the sprayability of such powder could be obtained by performing the heat treatment in argon atmosphere and using a slower cooling rate than used for F-HT 875 °C-4.

### Effect of Coating Heat Treatment

The best coating, i.e., the coating produced with the fine powder modified under argon (F-HT 875 °C-4), was heat treated to be more representative of an in-service state.

**Fig. 15:**
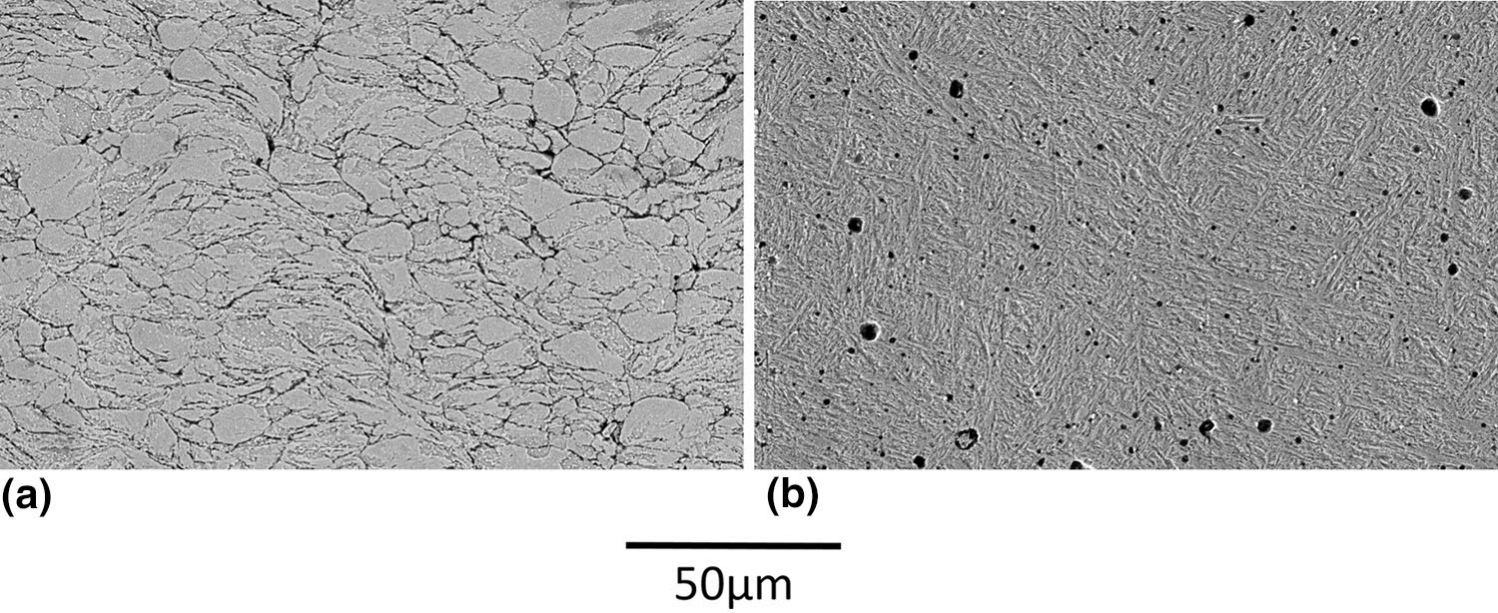
Etched microstructures (SE, 15kV, 1000X) of deposits produced with fine powder modified under argon (a) As-Sprayed (b) after post spray heat treatment

Figure 15 shows the etched microstructures of the deposit before and after heat treatment. For the as-sprayed coating, the boundaries of the deposited splats are clearly visible after etching, indicating imperfect particle bonding. Contrary, these boundaries are not visible after heat treatment at 1200 °C, demonstrating sintering of the particles at high temperature. The pores shown in the micrograph after heat treatment are believed to be created during the sintering process, when poorly bounded interfaces break into isolated pores, followed by pore coalescence, coarsening and rounding due to surface diffusion (Ref 37). The microstructure of the heat treated deposit is composed of lenticular features indicative of martensite (Ref 30), while carbides, if present, are too small to be observed.

Heat treated deposit hardness was measured at 56.2 HRC, which is comparable to bulk tempered H13 at 38-53 HRC (Ref 31). The deposit microstructure and hardness demonstrate that the cold sprayed H13 deposits are heat treatable and confirm their great potential for several applications.

## Conclusions

In this study, a novel powder modification method based on powder heat treatment in a rotary tube furnace was developed to drastically improve the cold sprayability of H13 tool steel powder. In the as-received condition, coarse powder (d_50_: 36.9 µm) could not be deposited while low coating deposition rate and quality were obtained for a finer powder (d_50_: 7 µm). Powder heat treatment of the coarse powder led to a 60% reduction in powder hardness, which was sufficient to allow deposition of a cracked coating. Powder heat treatment of the fine powder allowed simultaneous annealing and agglomeration of smaller particles, which was demonstrated to be beneficial for deposition rate and coating quality as sound coatings were deposited. Powder heat treatment temperature, cooling rate and protective atmosphere exempt of oxygen were found to be key parameters governing cold sprayability. With the aim of increasing the heat treatment process productivity, the cooling rate in nitrogen atmosphere was augmented from 22 °C/h (typical for cooling bulk H13) to 350 °C/h, with some extent of detrimental effects observed on deposition rates due to increased critical velocities linked to less effective powder softening. Using a HP argon atmosphere, the best coating quality with DE of ~70% was obtained for a powder heat treated at 875 °C at 350 °C/h cooling rate. It is possible that further gains can be obtained by further tailoring the initial powder particle size or further improving heat treatment conditions, such as cooling rates. Finally, it was found that H13 deposit could effectively be processed by hardening and tempering heat treatment in order to obtain a final hardness of 56.2 HRC equivalent to bulk H13.
